# Innate positive chemotaxis to pollen from crops and banker plants in predaceous biological control agents: towards new field lures?

**DOI:** 10.1038/srep12729

**Published:** 2015-08-03

**Authors:** Shu Li, Xiaoling Tan, Nicolas Desneux, Giovanni Benelli, Jing Zhao, Xinhai Li, Fan Zhang, Xiwu Gao, Su Wang

**Affiliations:** 1 Institute of Plant and Environment Protection, Beijing Academy of Agriculture and Forestry Sciences, Beijing, 100097, China; 2 Department of Entomology, China Agricultural University, Beijing 100193, China; 3 Institute of Zoology, Chinese Academy of Sciences, Beijing, 100101, China; 4 French National Institute for Agricultural Research (INRA), UMR1355-ISA, 06903 Sophia-Antipolis, France; 5 Department of Agriculture, Food and Environment, University of Pisa, 56124 Pisa, Italy

## Abstract

Predator-prey interactions form the core of biological control of arthropod pests. Which tools can be used to monitor and collect carnivorous arthropods in natural habitats and targeted crops? Eco-friendly and effective field lures are urgently needed. In this research, we carried out olfactometer experiments assess innate positive chemotaxis to pollen of seven crop and banker plant by two important predatory biological control agents: the coccinellid *Propylea japonica* (Thunberg) and the anthocorid *Orius sauteri* (Poppius). We compared the attractiveness of pollens from crops and banker plants to that of common prey homogenates (aphids and thrips, respectively). Attractiveness of the tested odor sources was checked via field trapping experiments conducted in organic apple orchards and by release-recapture assays in organic greenhouse tomato crops. Maize and canola pollen were attractive to both *P. japonica* and *O. sauteri*, in laboratory and field assays. *P. japonica* was highly attracted by balm mint pollen, whereas *O. sauteri* was attracted by alfalfa pollen. Our results encourage the use of pollen from crops and banker plants as low-cost and eco-friendly attractors to enhance the monitoring and attraction of arthropod predators in biological control programs.

Predator-prey interactions form the core of biological control of arthropod pests, which have proven invaluable in the fields of both agricultural pest management and natural resource conservation^1^,^2^,^3^,^4^,^5^,^6^. Reductions in the costs of labor, pesticides, and equipment, as well as a possible return to pre-invasion ecological services, constitute a persuasive argument for enhancing conservational biological control in the years to come^7^,^8^,^9^. Bokonon-Ganta evaluates economic of biological control of mango mealybug, resulting in a benefit-cost ratio of 145:1^10^,^11^. That is far out weighing the cost of unsuccessful biological control program^12^,^13^,^14^,^15^. However, attention should be paid to the possibility of interactions with non-target organisms^16^,^17^. To reduce or avoid such interactions, new tools have been developed, including host specificity testing (e.g.^18^,^19^), which should be carried out in the target habitat whenever possible^20^,^21^. Today, large numbers of predatory arthropods are collected, mass-reared and then released in targeted agro-ecosystems in augmentative biological control programs^8^,^22^. These programs benefit from tools that can be used to attract, collect and monitor predatory arthropods in natural habitats and targeted crops. To meet this need, efforts have been made to shed light on the chemical and physical cues used for prey location by biological control agents^23^,^24^,^25^,^26^,^27^. Much emphasis has been dedicated to prey-source semiochemicals, including herbivore-induced plant volatiles, prey sexual pheromones, aggregation pheromones, and enemy-avoidance kairomones^28^,^29^,^30^,^31^,^32^,^33^,^34^,^35^. These semiochemicals have been proposed as field lures to be used in IPM programs to monitor natural enemy populations or to re-establish predator-prey relationships that have become decoupled in disturbed agricultural habitats^27^,^36^.

Olfactory cues produced by herbivore-free plants, such as volatile organic compounds from pollen and nectar also play a role in predatory arthropod attraction^33^. This phenomenon has been studied in many parasitoid species^37^,^38^, however little knowledge is available about the attractiveness of floral origin olfactory cues to predatory species^39^,^40^,^41^. In addition, a number of flowering plant species support predator arthropod populations by providing supplementary food (e.g. nectar and pollen)^37^,^40^, shelter for mating activity^9^,^42^,^43^ and protection from pesticides^44^,^45^,^46^. Food web enhancements using banker plants may help limit pest populations by re-attracting natural enemies, and diminishing the cost^47^,^48^,^49^.

Recent research on the harlequin ladybird, *Harmonia axyridis* (Pallas) showed that individuals tend to aggregate in close proximity to plant volatiles in their natural habitat^50^,^51^,^52^,^53^,^54^, and that abundant ladybird populations can be found in alfalfa and white clover fields during the entire growing season, seeking aphids or other Homoptera species^55^,^56^. Preliminary our observations have indicated that pollen of different crops (e.g. alfalfa, maize, and canola) attracts a number of predatory ladybirds and flower bugs during the early spring. Conserved native grassland flora located nearby orchards has been associated with increased predator biodiversity and enhanced biological control effectiveness^9^,^57^,^58^.

Based on these findings, we hypothesized that substances such as pollen from crops and banker plants will attract predator. We performed olfactometer experiments to assess innate positive chemotaxis toward seven crop and banker plant pollens by two important predators, the coccinellid *Propylea japonica* (Thunberg) and the anthocorid *Orius sauteri* (Poppius). In their native regions, both *P. japonica* and *O. sauteri* help to suppress outbreaks of various arthropod pests, such as aphids, thrips, and spider mites in orchards, fields and greenhouses^59^,^60^,^61^,^62^,^63^. We compared the attractiveness of different pollens from crops and banker plants to that of prey homogenates of *P. japonica* and *O. sauteri* (i.e. aphids and thrips, respectively). Attractiveness of the tested odor sources under field conditions was studied by field trapping experiments conducted in organic apple orchards and release-recapture assays in organic greenhouse tomato crops.

## Materials and Methods

### Predatory biocontrol agents

From May to June, 532 adults of *P. japonica* (213 males and 319 females) were collected from alfalfa fields, and 119 adults of the flower bug *O. sauteri* (61 males and 58 females) were collected from white leaf clover (*Trifolium repens* L.) fields, in Beiliu village, Changping, Beijing (N40°17′, E116°0′). Both predators were transferred to the laboratories of the Institute of Plant and Environment Protection (Beijing Academy of Agriculture and Forestry Sciences) for rearing. Predators were reared in cages measuring 40 × 40 × 45 cm, constructed of aluminum frames and 178 um mesh net fabric at a density of 30 pairs per cage. *P. japonica* and *O. sauteri* were provided *ad libitum* with 3^rd^ instar nymphs of *Megoura japonica* (Matsumura) aphids on horsebean (*Vicia faba* L.) and 3^rd^ instar nymphs of *Frankliniella occidentalis* (Pergande) thrips on cotton plant (Gossypium), respectively. Cages were placed into artificial environmental chambers (MH350, Sanyo, Japan) with T = 25 °C, RH = 65%, photoperiod (L:D) = 16:8 and light intensity = 900 lux.

### Olfactometer

**Figure 1:**
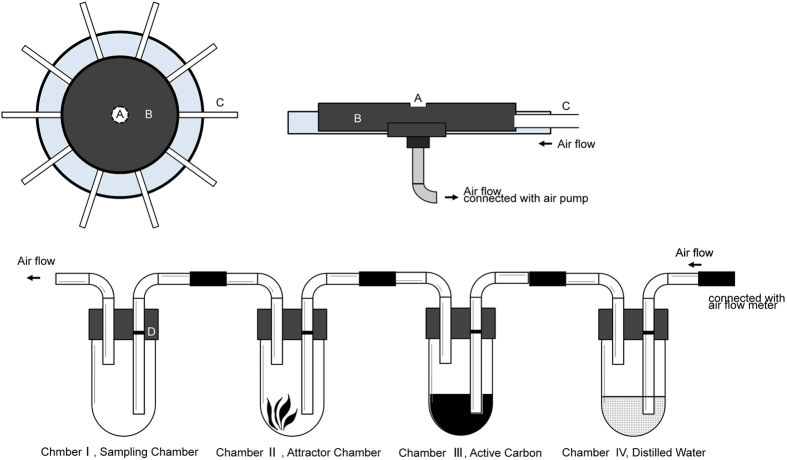
Ten-arm olfactometer used to test innate attraction to crop and banker plant pollen and prey homogenate in the predatory biological control agents *Propylea japonica* and *Orius sauteri*. A = entrance; B = release chamber for predator; C = arm. Airflow in from the arms is created by an air pump placed under the release chamber. For each arm, odor source is located in the attractor chamber. The connection between the attractor and sampling chambers is depicted (lateral view) at the bottom.

A custom-made ten-arm olfactometer (Jingyi, Beijing, China) was used to evaluate innate - chemotaxis of *P. japonica* and *O. sauteri* adults to odor sources of plant or insect origin. The olfactometer is described in Fig. 1. The entrance hole (diameter 3.0 cm) was located in the center of an insect release chamber (diameter = 45.0 cm, height = 1.5 cm), which was connected to 10 odor-source arms made of glass tubes (diameter = 0.5 cm, length = 3.0 cm). Air flow into each arm passed through a series of four chambers, included a distilled water filter chamber, an activated carbon filter chamber, an attractor chamber and a sampling chamber (for collecting attracted predators) (Fig. 1). At the sample movement chamber, the airflow meter was connected to the sampling chamber in each odor-source arm. An air pump (Sunny Industry, AirPower 6-CL, Beijing, China) was connected to the insect release chamber and set for constant airflow of 1,500 ml min^−1^^64^. To avoid any bias from surroundings, the 10-arm olfactometer was placed inside a white plastic box (120 × 120 × 80 cm) illuminated by four white, overhead LED white lights (800 lux each). Preliminary trials using empty odor-sources did not show directional bias for either predator (*P. japonica χ*^*2*^ = 7.131, *P* = 0.117; *O. sauteri*: *χ*^*2*^ = 8.379, *P* = 0.1196).

### Pollen collection and insect-borne odor-sources

Nine odor sources were evaluated for innate positive chemotaxis in *P. japonica* and *O. sauteri*. We tested seven floral-borne odor sources: canola pollen (*Brassica campestris* L. cv. Jingyou-1), maize pollen (*Zea mays* L. cv. Denghai-605), rose pollen (*Rosa chinensis* Jacq.), amaranth pollen (*Myosotis sylvatica* L.), white clover pollen (*Trifolium repens* L.), balm mint pollen (*Mentha haplocalyx* Briq.) and alfalfa pollen (*Medicago sativa* L.). The pollens were produced and purified at Zhongnong Company from China academy of agricultural science. All pollens were collected daily by shaking the flower in a plastic bag. The collected pollen was air-dried at room temperature for 48 h and subsequently passed through a screen to remove anthers and contaminants. Pollen collected from each flower was pooled and stored at −80 °C until used.

The two insect-borne odor-sources were homogenates of 3^rd^ instar nymphs of *M. japonica* and *F. occidentalis*, common preys of *P. japonica* and *O. sauteri*, respectively. Control odor source was clean air.

### Olfactometer experiments

In olfactometer experiments, 65 *P. japonica* female adults or 65 *O. sauteri* female adults were gently transferred into the release chamber before starved 12 hours. The nine odor sources, balanced for weight (50.0 mg/odor source) were placed into attractor chambers positioned at the end of each odor arm, with the last attractor chamber empty after 30 min, the insects found in the sampling chamber of each arm were counted. The test was replicated 10 times with each predator. In each replicate, odor sources were randomized and all olfactometer arms were rotated by 36° to avoid experimental bias. After each replicate, olfactometer components were cleaned by the soap-water rinse hexane method described in Benelli *et al*.^34^.

### Field attraction assays in organic apple orchard

We used the sampling trap described by Wang *et al*.^52^ to evaluate the attraction of *P. japonica* and *O. sauteri* toplant pollens (canola, maize, rose corolla, amaranth corolla, white clover, balm mint and alfalfa) in organic apple orchards (a natural habitat for both species). Prey homogenates of *M. japonica* and *F. occidentalis* were also tested in the orchard. A potential attractor, balanced for weight (100 g/trap), was placed into each sampling trap. Moist cotton (100 g) served as control^52^. The field survey was carried out in an organic apple orchard (WANG J-Y organic apple Co. Ltd. N40°10′, E116°2′, WANG J-Y village, Changping, Beijing) during August and September 2012. For each predator species, the test was repeated 10 times. In each replicate, field attraction was evaluated by placing 3 traps per treatment in the orchard. Sampling traps for each attractor or control were randomly distributed in the orchard at a height of 1.5 m above the ground. The minimum distance separating traps is 12 m that not to interfere with each other. After 24 h, for each species, the number of trapped adults was recorded.

### Release -recapture assays in organic greenhouse tomato crops

These experiments were carried out from May to July 2013 in organic tomato greenhouses (7500 cm × 600 cm each) at NOYA Organic Farm (N40°108′, E116°99′) in Pinggu County, Beijing. Initially, 1500 tomato plants, each with 6 main leaves, were transplanted into each greenhouse. After 5 days, over 500,000 spider mite adults, *Tetranychus cinabarinus* were released into the greenhouse. Eleven days later, randomly selected unmated *P. japonica* (35 adults) or *O. sauteri* (90 adults) were released in the greenhouse, when the spider mites get even distribution. We used the same sampling traps described above for our field attraction assays in organic apple orchards^52^. Ten sampling traps baited with different attractors (100 g each, control: moistened cotton) were set in each greenhouse 24 h post-release. After 3 days, the traps were inspected and the *P. japonica* and *O. sauteri* individuals were counted. The greenhouse tests were replicated 10 times. The site of each sampling trap was randomized in each replicate.

## Data analysis

Statistical analyses from both data form olfactometer and field tests were based on a log-linear model for inter-comparison group difference, in difference with the method described by Turling *et al*.^65^, Tamo *et al*.^66^ and Davison & Ricard^67^,^68^. We used on a stochastic model developed from Tamo *et al*.^66^, to allowing for data on the multinomial distribution. The number of predators choosing the *i*th arm (*i* = 1,…,10) indicates the relative attractiveness of the corresponding odor source, which is parameterized by λ_i_ . If only the odor source affects predator’s choice, and this effect is the same for all predators, the corresponding model is


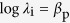


where β_p_ measures the attractive of either an empty arm or of the odor source in the *i*th arm. The ration of residual deviance to degree of freedom was less than 1, indicated therefore no data over dispersion.

## Results

### Innate positive chemotaxis in the olfactometer

**Figure 2:**
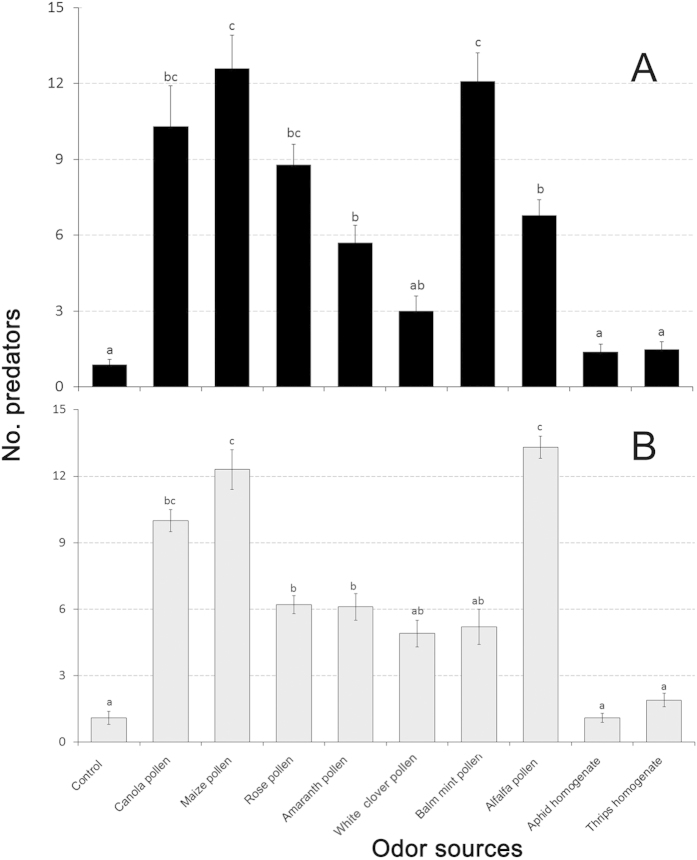
Behavioral responses of two predatory biological control agents. (**A**) *Propylea japonica* and (**B**) *Orius sauteri*, to various plant- and prey-borne substances in olfactometer tests. T-bars are standard errors. Bars having no letters in common represent significantly different response levels with each graph.

There were significant differences in the attractiveness of crop and banker plant pollens for predatory. In *P. japonica* experiments, canola pollen, maize pollen and balm mint pollen were more attractive compared to other treatments (*P* < 0.01). Rose corolla pollen, amaranth corolla pollen, white clover pollen and alfalfa pollen where also more attractive than the control (Fig. 2A). In *O. sauteri* experiments, canola pollen, maize pollen and alfalfa pollen were more attractive compared to other treatments (*P* < 0.01). Rose corolla pollen, amaranth corolla pollen, white clover pollen and balm mint pollen were more attractive than the control (Fig. 2B). Prey homogenate odors were not attractive.

### Field attraction in organic apple orchards

**Figure 3:**
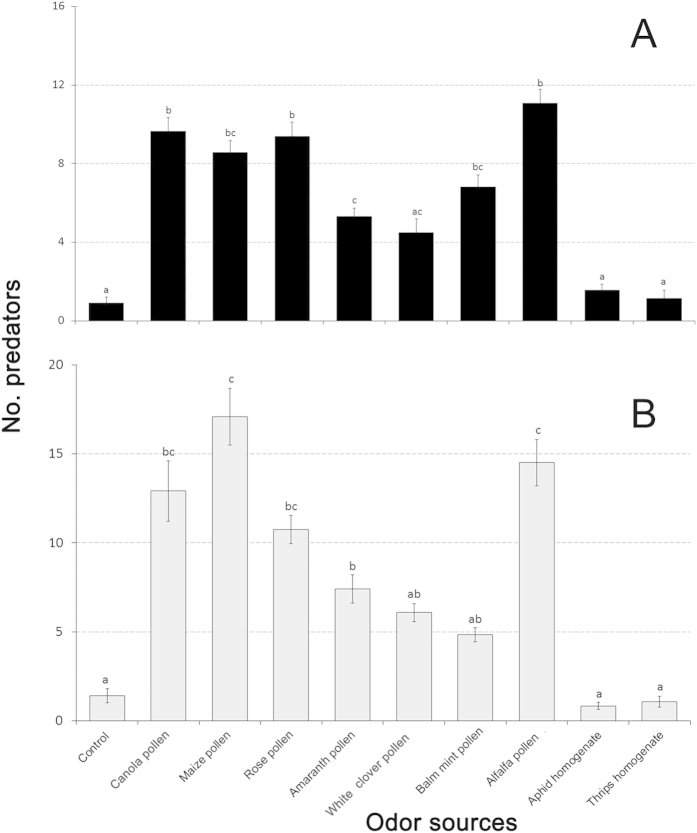
Attraction shown by two predatory biological control agents. (**A**) *Propylea japonica* and (**B**) *Orius sauteri*, to various plant- and prey-borne substances in organic apple orchards. T-bars are standard errors. Bars having no letters in common represent significantly different response levels with each graph.

Field attraction assays confirmed the behavioral responses seen in olfactometer experiments (Fig. 3). Balm mint pollen and maize pollen attracted more *P. japonica* than other treatments (*P* < 0.01), while rose corolla pollen, amaranth corolla pollen, white clover pollen and alfalfa pollen were more attractive than the control (Fig. 3A). Alfalfa pollen, maize pollen and canola pollen attracted more *O. sauteri* adults over other treatments (*P* < 0.01). Rose corolla pollen, amaranth corolla pollen, white clover pollen and balm mint pollen were more attractive than the control (Fig. 3B). Attraction to prey homogenates did not differ from the control.

### Release-recapture in organic greenhouse tomato crops

**Figure 4:**
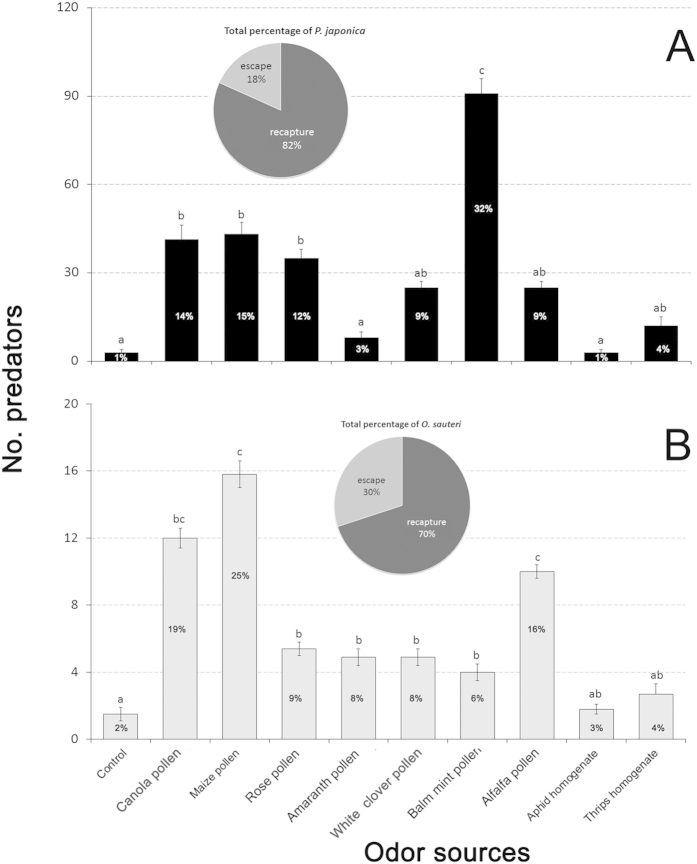
Post-release re-attraction of two predatory biological control agents. (**A**) *Propylea japonica* and (**B**) *Orius sauteri*, to various plant- and prey-borne substances in organic greenhouse tomato. T-bars are standard errors. Bars having no letters in common represent significantly different response levels with each graph. The pie showed total percentage of predators. The percentage of predators in bars is the ratio of attracted by each odor source predators to total recapture predators.

Release-recapture assays carried out with *P. japonica* and *O. sauteri* in greenhouse tomato crops showed results comparable to our previous experiments (Fig. 4). In *P. japonica* trials, balm mint pollen was the best lure, followed by maize pollen, canola pollen and rose corolla pollen (*P* < 0.001) (Fig. 4A). *P. japonica* attraction to amaranth pollen, aphid or thrip homogenates was not different from the control (Fig. 4A). In *O. sauteri* trials, maize pollen was the most effective lure, followed by canola pollen and alfalfa pollen (*P* < 0.001) (Fig. 4B). Rose corolla pollen, amaranth corolla pollen and white clover showed higher attraction than control or the prey homogenates (Fig. 4B). The majority of *P. japonica* (82%) and *O. sauteri* (70%) were recaptured.

## Discussion

For decades, researchers have sought effective lures to attract entomophagous insects from natural habitats into crops, since this could help reduce the need for natural enemy augmentation that relies on artificial mass rearing^8^,^20^,^52^. Traps enhanced with low-cost lures within an agro-ecosystem may help to monitor the population dynamics of released predatory insects, and could lead to decreased ecological risks resulting from their over-aggregation. However, experimental evidence of these dynamics for predaceous biological control agents is hard to find. Furthermore, mass rearing of natural enemies are not only high cost in China, but also faced quality control challenges^69^. However, inundative and seasonal inoculative releases of natural enemies are mass applied in greenhouse. The number of natural enemies available has increased dramatically in future. These programs benefit from tools that can be used to attract, collect and monitor predatory arthropods in natural habitats and targeted crops.

Crop and banker plant pollens have been investigated as alternative foods for predatory arthropods^39^,^40^,^41^. Although many studies about available on the cues guiding predatory biocontrol species towards these food sources, our results point out the important role for olfactory stimuli. We observed that crop and banker plant pollens are able to induce positive chemotaxis by Coccinellidae and Anthocoridae species in laboratory conditions, field orchard assays and release-recapture greenhouse experiments. On the other hand, crushed preys were poorly attractive to predaceous arthropods, this may be due to the fact that predators may have adapted to easier detect plant-borne olfactory cues associated with the herbivore’s presence rather than to the cues from the herbivore itself.

Interestingly, maize and canola pollen show good attraction rates for *P. japonica* and *O. sauteri*, in both laboratory and field assays. *P. japonica* is also attracted by balm mint pollen, while *O. sauteri* is attracted by alfalfa pollen^45^,^70^,^71^,^72^,^73^. We found that maize pollen is more attractive than canola pollen for both *P. japonica* and *O. sauteri*. This could be due to the high sugar content of maize pollen, which far exceeds that of canola pollen^38^. Indeed, sugar-rich food can enhance flight and the host foraging ability of predatory insects^74^, and may positively affect lifespan^73^,^75^,^76^. In the field, many species of predators can be less abundant in nectar-less cotton fields than in nectared ones^77^.

Previous research has shown that, in addition to sugars, VOC profiles from corn plants contain complex odor components including green leaf alcohols, aldehydes, derivate esters, terpenes, and sesquiterpenes^78^. Predatory insects may use these compounds as cues when foraging for prey^79^,^80^. For example, the 12-spotted ladybird *Coleomegilla maculata* (DeGeer) and the green lacewing *Chrysoperla carnea* (Stephens) showed electroantennographic responses to VOCs from corn pollen and leaves^81^. Also, balm mint plants are attractive to a number of herbivorous arthropods, including aphids, whiteflies and thrips^82^. Electrophysiological assays are required to shed light on which compounds are the principal sources of this attractiveness. Alfalfa pollen has been observed as one of the best lures for *O. sauteri*. Alfalfa has been already tested as an intercrop in cotton fields to aggregate predatory arthropods. When compared with pure cotton cultivation, the species richness index and diversity of natural enemies is often higher in intercropped fields^83^. At the time of alfalfa cutting, ladybirds, spiders and lacewings may move to cotton plants, helping to control outbreaks of *Aphis gossypii* Glover^84^. In addition, alfalfa fields frequently provide a favorable environment for the reproduction of aphidophagous coccinellids (e.g. *Coccinella septempunctata* L.)^85^ and flower bugs^83^. In the greenhouse release-recapture experiments, pollen attractiveness was lower compared to the results in the olfactometer and field assays in apple orchards. This may be due to other factors which affect the predator’s searching for non-prey foods. For instance, if greenhouse conditions presented higher prey densities than the other two experiment; this may deter the predators from utilizing non-prey food sources^41^. However, balm mint and maize pollen can still be proposed as good lures for *P. japonica*, and *O. sauteri*, respectively in greenhouses, since our results show positive chemotaxis to these two lures.

Overall, this research suggests that pollen from four plant species can serve as an eco-friendly and effective field lure for predatory biological control agents, both in orchard trapping surveys and in greenhouse release- reattraction assays. Maize, canola and balm mint pollens can be used for the coccinellid *P. japonica*, while maize, canola and alfalfa pollens are attractive to the flower bug *O. sauteri*. These results encourage the use of pollen from crops and banker plants as low-cost and eco-friendly attractors to enhance monitoring and attraction strategies in predatory arthropod-based biocontrol programs. The appropriate placement of suitably designed and improved sampling traps may prove to be a viable tactic for collecting predators.

## Additional Information

**How to cite this article**: Li, S. *et al*. Innate positive chemotaxis to pollen from crops and banker plants in predaceous biological control agents: towards new field lures?. *Sci. Rep*. **5**, 12729; doi: 10.1038/srep12729 (2015).
