# Corrigendum: Innate positive chemotaxis to pollen from crops and banker plants in predaceous biological control agents: towards new field lures?

**DOI:** 10.1038/srep44516

**Published:** 2017-03-16

**Authors:** Shu Li, Xiaoling Tan, Nicolas Desneux, Giovanni Benelli, Jing Zhao, Xinhai Li, Fan Zhang, Xiwu Gao, Su Wang

Scientific Reports 5: Article number: 1272910.1038/srep12729; published online: 08 03 2015; updated: 03 16 2017

The Acknowledgements section in this Article is incomplete:

“This research is supported by the National Basic Research Program of China (973 Program) (grant 2013CB127605), the Special Fund for Agroscientific Research in the Public Interest (grant nos 201303024 and 201303108), the Beijing NOVA program (No. Z121105002512039), and the Beijing municipal Science Foundation for Post doctor and Postdoctoral Foundation of Beijing Academy of Agriculture and Forestry Sciences”.

Should read:

“This research is supported by the National Basic Research Program of China (973 Program) (grant 2013CB127605), the Special Fund for Agroscientific Research in the Public Interest (grant nos 201303024 and 201303108), the Beijing NOVA program (No. Z121105002512039), the Beijing municipal Science Foundation for Post doctor and Postdoctoral Foundation of Beijing Academy of Agriculture and Forestry Sciences, and the Youth Funds of Scientific Research of BAAFS (QNJJ201410)”.

